# Intra-pericardial thrombin injection as bailout strategy in iatrogenic pericardial tamponade

**DOI:** 10.1007/s12471-022-01701-y

**Published:** 2022-06-01

**Authors:** D. Rottländer, M. Saal, H. Degen, M. Haude

**Affiliations:** 1 Department of Cardiology, Rheinlandklinikum Neuss, Neuss, Germany; 2 Department of Cardiology, Krankenhaus Porz am Rhein, Cologne, Germany; 3 Department of Cardiology, Faculty of Health, School of Medicine, University of Witten/Herdecke, Witten, Germany

**Keywords:** Pericardial tamponade, Thrombin, Cardiac tamponade, Coronary perforation, Percutaneous intervention

## Abstract

**Background:**

Cardiac tamponade is a rare but life-threatening complication of cardiac interventions. Despite prompt pericardiocentesis, clinical management can be challenging and sometimes haemodynamic stabilisation is difficult to achieve. Intra-pericardial thrombin injection after pericardiocentesis promotes haemostasis and acts as a sealing agent, as previously described for left ventricular free-wall rupture. We aimed to evaluate intra-pericardial thrombin injection as a bailout strategy for pericardial tamponade following percutaneous cardiac interventions.

**Methods:**

In a 5-year single-centre retrospective analysis we identified 31 patients with cardiac tamponade due to percutaneous intracardiac procedures. Intra-pericardial thrombin injection as a bailout strategy was administered in 5 of 31 patients (16.1%).

**Results:**

Patients receiving intra-pericardial thrombin were in a more critical state when thrombin was applied, as demonstrated by a higher rate of resuscitation (40% versus 26.9%) and a trend toward a prolonged stay in the intensive care unit (177.6 ± 84.0 vs 98.0 ± 31.4 h). None of the patients with pericardial tamponades treated with intra-pericardial thrombin needed cardiothoracic surgery. Mortality after 30 days was lower with intra-pericardial thrombin injection than with standard treatment (0% vs 15.4%). We observed no complications using intra-pericardial thrombin.

**Conclusion:**

Intra-pericardial thrombin injection could be considered as a bailout strategy for patients with iatrogenic pericardial tamponade due to percutaneous procedures. We recommend further evaluation of this technique in the clinical management of refractory pericardial tamponade.

**Supplementary Information:**

The online version of this article (10.1007/s12471-022-01701-y) contains supplementary material, which is available to authorized users.

## What’s new?

- Iatrogenic cardiac tamponade is a serious and life-threatening complication of cardiac interventions.
- This is the first study to investigate the outcome and clinical course of patients with pericardial thrombin administration as a bailout strategy in cases of cardiac tamponade.
- Patients with intra-pericardial thrombin injection were in a more critical state, but cardiothoracic surgery could be avoided in all cases. Also, 30-day mortality was lower than in the control group.

## Introduction

The number and complexity of percutaneous cardiac interventions is increasing. Most procedures need anticoagulation to prevent thrombotic events. Furthermore, trans-septal puncture or left atrial access is mandatory in some cardiac interventions. Therefore, pericardial effusion (PE) and tamponade are well-known and potentially fatal complications of percutaneous cardiac interventions, such as closure of a patent foramen ovale, percutaneous coronary intervention (PCI), percutaneous heart valve repair (e.g. transcatheter aortic valve replacement or transcatheter mitral valve repair), left atrial appendage closure and placement of a pacemaker or an implantable cardioverter-defibrillator (ICD) [1, 2]. Procedure-related PE is the result of a perforation and develops rapidly in most cases. Early diagnosis using transthoracic (TTE) or transoesophageal (TOE) echocardiography and prompt treatment of PE is lifesaving [3, 4]. Some tamponades require urgent resuscitation with pericardiocentesis and continued evacuation using an indwelling catheter. In some cases, percutaneous drainage may not successfully resolve the tamponade and urgent cardiothoracic surgery may be required. As transport time to the next available cardiac surgery may vary due to local conditions, bailout strategies are necessary to reduce complication-related mortality.

Thrombin promotes haemostasis by converting fibrinogen to fibrin and therefore thrombosis. Iatrogenic arterial aneurysms following interventions via the femoral artery are, if suitable, treated using local thrombin injection [5, 6]. For left ventricular free-wall rupture in patients with acute myocardial infarction, fibrin glue injected intra-pericardially was reported as a non-operative treatment strategy [7–9]. Also, direct thrombin administration showed a favourable outcome in this setting as described in a previously published case report [10]. Intra-pericardial thrombin application in patients with iatrogenic PE as a bailout strategy for haemodynamically unstable patients has not been reported to date. Therefore, we report on intra-pericardial thrombin injection as a bailout strategy and compared 30-day mortality and clinical course with those of non-thrombin-treated controls.

## Methods

### Study cohort

This retrospective study was performed in compliance with the Declaration of Helsinki. Individual written consent was obtained from every patient. Due to the retrospective nature of our study no ethical approval was required. This single-centre study cohort of a large cardiovascular centre comprised 31 consecutive patients with cardiac tamponade due to percutaneous cardiac interventions between 1 January 2010 and 1 July 2015. In the case of peri-interventional cardiac tamponade the interventional team performed pericardiocentesis and complication management. Intra-pericardial thrombin injection was used as a bailout strategy in 5 patients (16.1%), with the decision made on an individual basis by the responsible interventionalists. Thrombin was administered in cases of refractory intra-pericardial bleeding and prolonged haemodynamic instability despite reversal of anticoagulation, medicinal circulatory support and ongoing treatment of the cause of the PE. In order to evaluate the efficacy and safety of intra-pericardial thrombin injection, we compared the clinical course of these 5 patients with that of the patients with cardiac tamponade without intra-pericardial thrombin administration.

### Peri-interventional cardiac tamponade

Peri-interventional PE was defined as acute fluid accumulation in the pericardium, resulting in compression of cardiac chambers verified by TTE or TOE. All patients had undergone preprocedural echocardiography, which ruled out PE prior to cardiac intervention. Typical echocardiographic signs of PE were diastolic right ventricular collapse, late diastolic right atrial inversion, septal shift, decrease of E/A ratio and plethoric inferior vena cava. Echocardiography was immediately performed in cases of suspected PE. Clinical signs of PE were chest pain, nausea, abdominal discomfort in combination with hypotension and tachycardia as well as sometimes bradycardia and dyspnoea. Furthermore, pulsus paradoxus was infrequently observed. An adjuvant marker of tamponade was straightening and immobility of the left heart border on fluoroscopy.

### Pericardiocentesis

Adequate local anaesthesia was administered if needed. In brief, a guidewire was inserted into the pericardial space under either fluoroscopy or echocardiographic control and a 6 or 7 French pigtail catheter was introduced over the guidewire. Finally, continuous negative pressure was applied to the pigtail to empty the pericardial space. Subcostal access was used successfully in all cases.

### Intra-pericardial thrombin injection

Intra-pericardial thrombin (Pfizer, Collegeville, PA, USA) injection was used as a bailout strategy when no haemodynamic stabilisation was achieved after pericardiocentesis and systemic reversal of anticoagulation using protamine, fresh frozen plasma and prothrombin complex concentrate. Thrombin was injected when intra-pericardial bleeding was not otherwise controllable and the last remaining option was cardiothoracic surgery. After emptying the pericardial space of blood by aspiration, 5000 IU thrombin mixed with 20 ml saline solution (250 units/ml) was injected into the pericardial space using the pigtail catheter or a sheath with a larger lumen. In the case of catheter obstruction, a new pigtail catheter was introduced into the pericardial space and again constant negative pressure was applied.

### Statistical analysis

Statistical analysis was performed using PASW statistics 18 software (SPSS, Chicago, IL, USA). All variables were tested for normal distribution with the Kolmogorov-Smirnov test. In the case of normal distribution, the results are given as mean ± standard error of the mean (SEM), otherwise as median and 95% confidence interval. Differences between groups and subgroups were evaluated by chi-square test for discrete variables and Student *t*-test for continuous variables. For ordinal data the Mann-Whitney U test was used. The Kaplan-Meier method was used to calculate 30-day mortality. A *p-*value < 0.05 was considered statistically significant.

## Results

A total of 31 patients with cardiac tamponade due to percutaneous cardiac interventions were included in this retrospective study. The demographic variables of the study population are shown in Tab. 1. Of the patients, 45.2% were male and 54.8% female. Mean age was 75.8 ± 1.8 years. Peri-interventional, haemodynamically relevant pericardial tamponade following pericardiocentesis occurred in 17 of 9530 screened patients undergoing PCI (0.2%), in 3 of 335 patients undergoing percutaneous valve repair (0.9%) and in 11 of 1485 patients undergoing device implantation (0.7%). Interventional treatment of chronic total occlusion involved a higher risk of pericardial tamponade (4 of 256 patients, 1.5%) than regular PCI (13 of 9274, 0.1%).

**Table 1:** Patient characteristics

	PT (*n* = 31)	
*Characteristic*	*n*	
Age	31	75.84 ± 1.8
Male	14	45.2%
NT-proBNP (pg/ml)	31	1584.74 ± 542.4
Left ventricular ejection fraction (%)	31	53.03 ± 2.1
*Cardiac intervention*		
Percutaneous coronary intervention	17	54.8%
– Chronic total occlusion	4	12.9%
– Rotablation	1	3.2%
Percutaneous valve intervention	3	9.6%
– Transcatheter aortic valve implantation	1	3.2%
– Edge-to-edge mitral valve repair	1	3.2%
– Indirect mitral annuloplasty	1	3.2%
Cardiac implantable electronic device	11	35.5%
– Pacemaker	10	32.3%
– Implantable cardioverter-defibrillator	1	3.2%
*Patient history*		
Arterial hypertension	30	96.8%
Diabetes mellitus	3	9.7%
Smoking	5	16.1%
Obesity	7	22.6%
Hyperlipidaemia	15	48.4%
Heart failure	3	9.7%
Chronic renal failure	7	22.6%
Atrial fibrillation	5	16.1%
*Medication (hospital admission)*		
Coumarin	5	16.1%
Direct oral anticoagulation	1	3.2%
Acetylsalicylic acid	24	77.4%
Clopidogrel	18	58.1%

*PT* pericardial tamponade, *NT-proBNP* N-terminal pro-B-type natriuretic peptide

Pericardial tamponade in patients undergoing PCI was predominantly associated with three-vessel coronary artery disease (10 of 17 patients, 58.8%). Only one case of pericardial tamponade associated with heart catheterisation occurred during laevocardiography. PCI of the right coronary artery (RCA) was complicated by pericardial tamponade in 7 patients, and pericardial tamponade was observed during PCI of the circumflex (Cx) artery (4 cases) and left anterior descending (LAD) artery (4 cases). The remaining affected vessels were intermediate and left main. In cardiac device implantation perforation of the right ventricle by a right ventricular lead was responsible for pericardial tamponade. In our cohort no patient suffered from cardiac tamponade due to right atrial or coronary sinus perforation during implantation of a pacemaker, ICD or cardiac resynchronisation therapy device (CRT). Perforating devices were predominantly the guidewire in the case of PCI and the right ventricular lead during device implantation. In one case the rotablator (Boston Scientific, Marlborough, MA, USA) was responsible for the perforation leading to pericardial tamponade. In percutaneous valve repair PE was caused by coronary sinus perforation during indirect mitral annuloplasty using the Carillon Mitral Contour System (Cardiac Dimensions, Kirkland, WA, USA) (*n* = 1), edge-to-edge mitral valve repair using MitraClip (Abbott Vascular, Menlo Park, CA, USA; *n* = 1) and transcatheter aortic valve replacement (*n* = 1).

### Intra-pericardial thrombin injection

Five patients (16%) received intra-pericardial thrombin injection as a bailout strategy in haemodynamically critical situations with refractory pericardial bleeding. Patient characteristics are displayed in Tab. 2. In these patients mean age did not differ significantly from that of the standard treatment group (74.8 ± 4.1 vs 76.0 ± 2.0 years). Intra-pericardial thrombin injection was applied in three cases of PCI complicated by perforation of the LAD, Cx and RCA. Furthermore, thrombin was administered in one case after coronary sinus perforation due to percutaneous indirect mitral annuloplasty and in one case after right ventricular perforation due to implantation of a right ventricular lead. A detailed description of all five cases is available in the Electronic Supplementary Material.

**Table 2:** Clinical characteristics of patients undergoing pericardial thrombin injection as a bailout strategy for peri-interventional cardiac tamponade

Patient	1	2	3	4	5	
*Characteristic*						
Age (years)	84	61	76	82	73	74.8 ± 4.1
Sex	Male	Male	Male	Male	Male	100%
*Cardiac intervention*						
Percutaneous coronary intervention		+		+	+	60%
– RIM					+	
– Cx		+				
– RCA				+		
– Chronic total occlusion				+		
Carillon Mitral Contour System			+			20%
Pacemaker implantation	+					20%
*Perforating device*						
Guidewire		+	+		+	60%
Balloon				+		20%
Right ventricular lead	+					20%
*Pericardial tamponade*						
Drained pericardial fluid (ml)	2000	375	2000	285	800	1092.0 ± 380.8
End-diastolic pericardial effusion (cm)	3	2.7	3.8	2.1	2.5	2.85 ± 0.3
Time to removal of pericardial drain (h)	72	96	72	48	24	62.4 ± 12.2
*Clinical course*						
Resuscitation	+		+			40%
Time in intensive care unit (h)	480	72	240	72	24	177.6 ± 84.0
Time to discharge from hospital (days)	32	5	10	6	4	11.4 ± 4.7
Intubation	+		+			40%
Duration of invasive ventilation (h)	480		170			
Catecholamines	+	+	+	+	+	100%
Sepsis	+					20%
Concentrated red cells (*n*)	4		3			
CVVH	+					20%
Cardiothoracic surgery	–	–	–	–	–	0%

*RIM* ramus intermedius,* Cx* circumflex artery, *RCA* right coronary artery, *CVVH* continuous veno-venous haemodialysis, *LAD* left anterior descending artery

### Comparison of pericardial thrombin injection with standard treatment

**Fig. 1:**
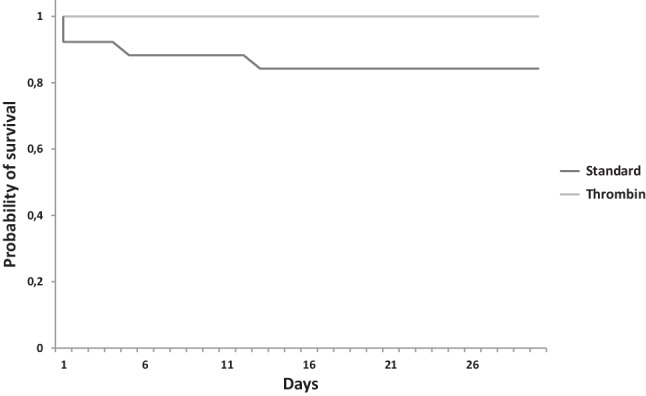
Mortality of patients with and without intra-pericardial thrombin administration for peri-interventional cardiac tamponade. Kaplan-Meier curve of 30-day survival probability after peri-interventional cardiac tamponade with (*n* = 5) or without (*n* = 26) intra-pericardial thrombin injection

**Fig. 2:**
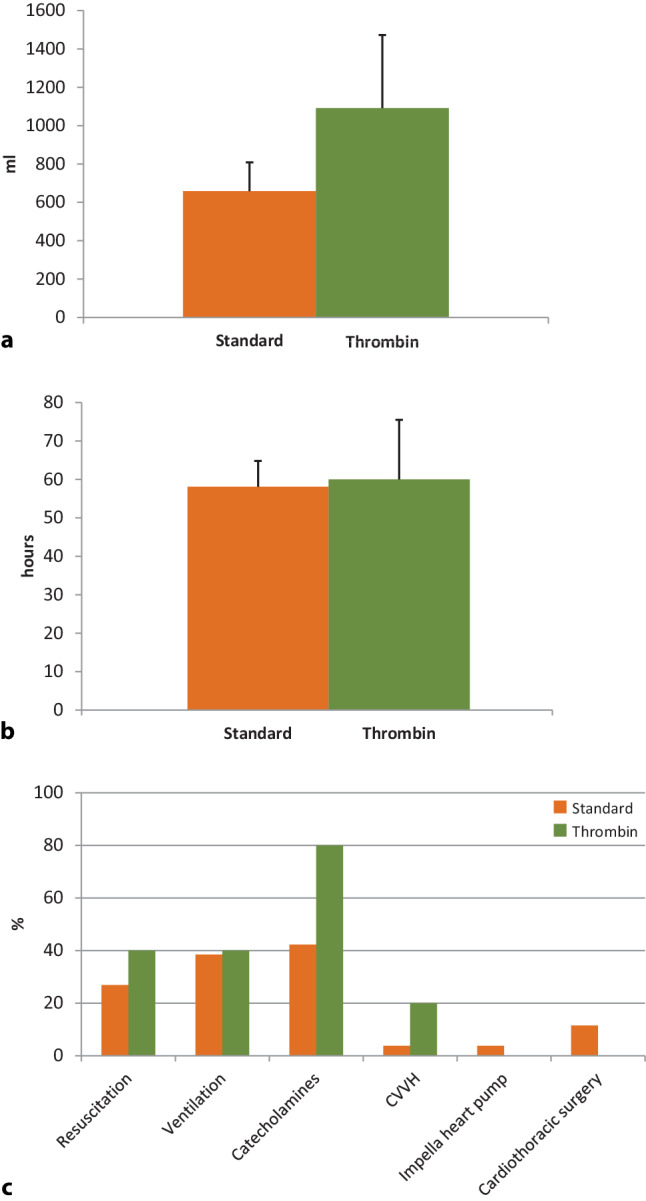
**a–c** Comparison of the clinical course of peri-interventional cardiac tamponade in patients with or without administration of intra-pericardial thrombin. **a** Comparison of drained pericardial fluid (millilitres) between standard treatment (*n* = 26) and pericardial thrombin application (*n* = 5). **b** Comparison of time to removal of pericardial drain (hours) between standard treatment and pericardial thrombin injection. **c** Percentage of specific treatments in clinical management of peri-interventional cardiac tamponade in the presence or absence of intra-pericardial thrombin application. *CVVH* continuous veno-venous haemodialysis

Mortality after 30 days was lower in patients with intra-pericardial thrombin injection compared to standard treatment (0 of 5 patients, 0% vs 4 of 26, 15.4%; Fig. 1). However, patients undergoing pericardial thrombin administration were in a more critical state when thrombin was injected into the pericardial space. This was demonstrated by a higher rate of resuscitation (2 of 5 patients, 40% vs 7 of 26 patients, 26.9%) and a trend toward a prolonged time in the intensive care unit (177.6 ± 84.0 h vs 98.0 ± 31.4 h). Furthermore, none of the patients with pericardial tamponades treated with intra-pericardial thrombin needed cardiothoracic surgery (0 of 5 patients, 0% vs 3 of 26 patients, 11.5%). Fig. 2 summarises the clinical course during the 30-day follow-up in patients with pericardial tamponade during percutaneous cardiac intervention treated with or without pericardial thrombin.

**Fig. 3:**
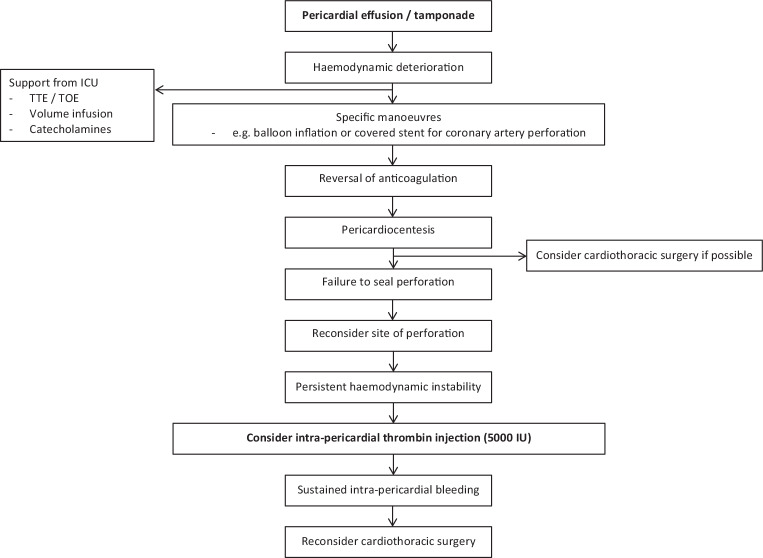
Iatrogenic pericardial tamponade workflow. *ICU* intensive care unit, *TTE* transthoracic echocardiography, *TOE* transoesophageal echocardiography

We observed no complications using intra-pericardial thrombin. In particular, no case of systemic application causing symptomatic embolism was observed. Furthermore, no treated patient reported symptoms of pericarditis following intra-pericardial thrombin administration. Fig. 3 proposes a workflow for iatrogenic cardiac tamponade with intra-pericardial thrombin administration as a bailout strategy prior to cardiothoracic surgery.

## Discussion

In this retrospective analysis we investigated the safety and efficacy of intra-pericardial thrombin injection as a bailout strategy in iatrogenic PE. The incidence of cardiac tamponade in percutaneous cardiac interventions varies depending on the type of procedure, technique of assessment and level of anticoagulation between < 1% and 6% [1]. Importantly, to date no prospective data are available, and small to moderate PE may be underreported. In cardiac pacemaker implantation the perforation rate within 7 days was reported to be 1.7% [11]. Cardiac effusion requiring pericardiocentesis was found in 1.5% of patients during implantation of an ICD [12]. Therefore, the incidence of haemodynamically relevant pericardial tamponade requiring pericardiocentesis in pacemaker, ICD or CRT implantation was low in our study population with an incidence of 0.7%. Coronary artery perforation in PCI has been reported to be between 0.19% and 0.35% [13, 14]. Perforation occurs more often during treatment of chronic total occlusion or rotablation. Guidewire-induced coronary perforation can be successfully treated by balloon inflation, vessel embolisation or covered stent implantation as standard treatment in most cases [15].We found peri-interventional, haemodynamically relevant pericardial tamponade followed by pericardiocentesis in 17 of 9530 patients undergoing PCI.

We observed one case of guidewire perforation of the coronary sinus during implantation of the Carillon Mitral Contour System, while the TITAN trial did not report a case of cardiac perforation [16]. In general, percutaneous heart valve repair showed an incidence of PE of 0.9%. All valve procedures with trans-septal puncture involve a risk of being complicated by cardiac tamponade.

Cardiologists routinely use thrombin for iatrogenic femoral pseudoaneurysms, which promotes thrombosis and stops extra-arterial bleeding [17]. Embolic events of the lower limb occur in approximately 2% when thrombin is used for closure of femoral pseudoaneurysms [17, 18]. However, most of the thromboembolic events are asymptomatic due to dilution and fibrinolysis of thrombin in the circulation. We evaluated the use of intra-pericardial thrombin administration as a bailout strategy for pericardial tamponade. In a case report, intra-pericardial thrombin injection was successfully used for treatment of left ventricular free-wall rupture following acute myocardial infarction [10]. For the first time, we used intra-pericardial thrombin application in cases of pericardial tamponade due to peri-interventional cardiac perforation. Compared to standard treatment more patients needed resuscitation and time in the intensive care unit was prolonged in the thrombin injection cohort. Also, the amount of drained pericardial blood was increased, indicating more severe and uncontrollable bleeding due to cardiac perforation in these patients. Mortality was lower in the thrombin injection group compared to standard treatment and no patient underwent cardiac surgery, while this was necessary in 11.5% of the standard treatment group. However, cardiac surgery might facilitate beneficial effects, e.g. by removing thrombus from the pericardial space. Also, the long-term outcome of intra-pericardial thrombin injection remains unclear, while 30-day follow-up revealed no side-effects of this emergency treatment. For centres without on-site cardiac surgery, we report the first evidence that intra-pericardial thrombin administration might be a feasible option.

In summary, we report for the first time about a new bailout strategy for iatrogenic cardiac tamponade. A retrospective approach was used, while prospective trials to study complications are not feasible. However, in our large interventional cardiovascular centre complication rates were low, but due to the large number of procedures we could identify 31 patients with pericardial tamponade, of whom 5 were treated successfully with intra-pericardial thrombin administration. Therefore, pericardial thrombin injection might be a new bailout strategy, which in this pilot study appears feasible, effective and safe. No cardiac or extracardiac embolism was observed and treatment success could be confirmed immediately.

### Study limitations

This study is of a retrospective, case-based nature and should be seen as hypothesis generating due to the small number of patients treated with intra-pericardial thrombin injection. Long-term adverse effects have to be investigated, as our retrospective data do not involve long-term follow-up. A prospective, randomised trial would be needed to verify our results. Prospective trials of complication management are difficult to perform, since randomisation is mostly impossible.

## Conclusion

Intra-pericardial thrombin injection might be a new bailout strategy for patients with iatrogenic pericardial tamponade due to percutaneous intracardiac procedures. Therefore, we recommend further evaluation of this technique in the clinical management of severe pericardial complications in the catheter laboratory.

## Supplementary Information

Detailed case descriptions of patients with intrapericardial thrombin administration.
