# Supplementary material for: Intra-pericardial thrombin injection as bailout strategy in iatrogenic pericardial tamponade
Source: Neth Heart J. 2022 Jun 1;31(2):61–7. doi: 10.1007/s12471-022-01701-y (PMC9892408; doi:10.1007/s12471-022-01701-y)
Supplement: Supplementary file 1 — Detailed case descriptions of patients with intrapericardial thrombin administration. [file 12471_2022_1701_MOESM1_ESM.docx]

**Supplementary material**

*Patient 1* was an 84 year old male who suffered from cardiac tamponade after temporary RV-lead placement (Supreme JSN catheter, Abbott, US) indicated by third degree AV-block. Concomitant RCA-PCI with implantation with two drug-eluting stents (DES) was performed. Due to hemodynamic relevant tamponade shortly after the procedure, the patient underwent resuscitation while pericardiocentesis was performed. In TTE the maximum diameter of the PE was 3 cm and RV rupture due to RV lead perforation was diagnosed. Intra-pericardial thrombin injection was used as a bailout strategy, while no hemodynamic stabilization was achieved after pericardiocentesis and reversion of anticoagulation using protamine, FFP and PCC. The patient also received four units of concentrated red cells (CRC). After thrombin administration hemodynamic stabilization was achieved and the patient was transferred to the intensive care unit (ICU). In total, 2000 ml of blood was drained from the pericardial space. After 72 hours the pericardial drainage was removed. The clinical course was complicated by sepsis, but the patient was discharged from ICU at day 20 and from hospital at day 32 after the index procedure.

*Patient 2* was a 61 year old male who was admitted to hospital with angina at ordinary physical activity (Canadian Cardiovascular Society (CCS) class III). Coronary angiography revealed two-vessel coronary artery disease with significant Cx stenosis and moderate mid-LAD stenosis. Guidewire perforation (Sion Blue, ASAHI Intecc, Japan) led to type III coronary artery perforation. The patient immediately became hemodynamically instable and pericardiocentesis was performed, while the balloon was inflated at the perforation site. In TTE the maximum diameter of circular pericardial effusion was 2.7 cm. After balloon deflation (20 minutes inflation time) coronary perforation persisted and the patient needed high doses of catecholamine. Covered stent was unable to cross the perforation site due to heavy calcification. While balloon inflation was performed again, intra-pericardial thrombin injection was used as a bailout strategy, while patient remained unstable. After thrombin administration, hemodynamic stabilization was achieved and after balloon deflation coronary perforation was absent. PCI was completed with an excellent angiographic result. The patient was transferred to ICU. In total, 375 ml of blood was drained from the pericardial space. After 96 hours the pericardial drainage was removed. The patient was discharged from ICU at day 3 and from hospital at day 5 after the cardiac tamponade.

*Patient 3* was a 76 year old male patient with moderate to severe secondary mitral regurgitation due to ischemic cardiomyopathy. The patient was scheduled by the local heart team for CS based indirect mitral annuloplasty using a Carillon mitral contour system. Due to a challenging course of the CS, a hydrophilic guidewire (Glidewire GR3506, Terumo Corporation, Japan) was difficult to place in the distal CS and multiple attempts were necessary. Thereby, the guidewire was advanced in an anterior sidebranch of the coronary sinus and CS was dissected. Afterwards, insertion of the hydrophilic guidewire into the distal CS was successful and the indirect mitral annuloplasty could be performed. Immediately after the procedure the patient was hemodynamically instable and showed PE in TTE. PE was most probably due to guidewire perforation of the CS. TTE revealed a hemodynamic relevant PE with an end-diastolic diameter of maximum 3.8 cm. Pericardiocentesis was immediately performed and in total 2000 ml blood was drained. Regardless of hemodynamic management including application of catecholamines, protamine, CRC and FFP, the patient needed resuscitation with invasive ventilation. As a bailout strategy, 5000 IU of thrombin was administered intra-pericardially after removing pericardial fluid leading to hemodynamic stabilization. As a consequence of thrombin administration, PE could be treated successfully. The procedure was terminated and the patient was transferred to ICU. After 72 hours the pericardial drain was removed. The patient was discharged from ICU at day 10 and transferred to a weaning unit at day 10 after index procedure.

*Patient 4* was an 82 year old male who was admitted to hospital with CCS class II angina for scheduled RCA CTO. Antegrade guidewire insertion over a heavily calcified coronary occlusion of medial RCA was performed. Dilatation with NC-balloon (ACCUFORCE 2.5 x 20 mm, Terumo Cooperation, Japan) led to a type III coronary artery perforation. The patient immediately became hemodynamically instable and pericardiocentesis was performed, while the balloon was inflated at proximal RCA. In TTE the maximal diameter of pericardial effusion was 2.1 cm. The patient needed high doses of catecholamine. While covered stent implantation was no option due to heavily calcified persistent coronary stenosis without appropriate lesion preparation, intra-pericardial thrombin injection was used as a bailout strategy. After thrombin administration, hemodynamic stabilization was achieved. The balloon in the proximal RCA was deflated with re-approved proximal RCA occlusion. The procedure was terminated. The patient was transferred to ICU. In total, 285 ml of blood was drained from the pericardial space. After 48 hours the pericardial drainage was removed. The patient was discharged from ICU at day 3 and from hospital at day 6 after performance of the CTO.

*Patient 5* was a 73 year old male who was admitted to hospital with CCS class III angina. Coronary angiography revealed one vessel coronary artery disease with significant intermediate stenosis and consecutive RIM-PCI with implantation of one DES was performed. Patient was transferred to the general cardiology ward. The patient became hemodynamically instable within one hour after the procedure. TTE revealed a PE and pericardiocentesis was performed. In TTE the maximum diameter of PE was 2.5 cm. Patient was hemodynamically instable and re-transferred to the cathlab. The patient needed high doses of catecholamine. Coronary angiography revealed distal guidewire (PT2 moderate support, Boston Scientific, US) perforation of the peripheral RIM and balloon inflation was immediately performed. After balloon deflation coronary perforation persisted. While covered stent implantation was no option in case of distal guidewire perforation, intra-pericardial thrombin injection was used as bailout strategy due to ongoing hemodynamic instability. Furthermore, thrombin was also administered in the distal RIM via a microcatheter (Corsair microcatheter, ASAHI Intecc, Japan) leading to iatrogenic RIM occlusion. Afterwards hemodynamic stabilization was achieved and RIM was occluded at the medial segment. The patient was transferred to ICU. In total, 800 ml of blood was drained from the pericardial space. After 24 hours the pericardial drainage was removed. The patient was discharged from ICU at day 1 and from hospital at day 4 after the cardiac tamponade.
